# Advances in Therapeutic Strategies for the Management of *Clostridioides difficile* Infection

**DOI:** 10.3390/jcm13051331

**Published:** 2024-02-26

**Authors:** Antonio Vitiello, Michela Sabbatucci, Andrea Zovi, Antonio Salzano, Annarita Ponzo, Mariarosaria Boccellino

**Affiliations:** 1 Ministry of Health, Directorate-General for Health Prevention, Viale Giorgio Ribotta 5, 00144 Rome, Italy; avitiello@hotmail.it (A.V.); a.salzano@sanita.it (A.S.); 2 Department Infectious Diseases, Italian National Institute of Health, Viale Regina Elena 299, 00161 Rome, Italy; 3 Ministry of Health, Directorate General of Hygiene, Food Safety and Nutrition, Viale Giorgio Ribotta 5, 00144 Rome, Italy; zovi.andrea@gmail.com; 4 Department of Biology and Biotechnology, University of Pavia, 27100 Pavia, Italy; annarita.ponzo01@universitadipavia.it; 5 Department of Precision Medicine, University of Campania “Luigi Vanvitelli”, 81100 Naples, Italy; mariarosaria.boccellino@unicampania.it

**Keywords:** *Clostridioides difficile*, CDI, healthcare-associated infection, antibiotics, monoclonal antibodies, phages, vaccines, FMT

## Abstract

The infection caused by *Clostridioides difficile* represents one of the bacterial infections with the greatest increase in incidence among nosocomial infections in recent years. *C. difficile* is a Gram-positive bacterium able to produce toxins and spores. In some cases, infection results in severe diarrhoea and fulminant colitis, which cause prolonged hospitalisation and can be fatal, with repercussions also in terms of health economics. *C. difficile* is the most common cause of antibiotic-associated diarrhoea in the healthcare setting. The problem of bacterial forms that are increasingly resistant to common antibiotic treatments is also reflected in *C. difficile* infection (CDI). One of the causes of CDI is intestinal dysmicrobialism induced by prolonged antibiotic therapy. Moreover, in recent years, the emergence of increasingly virulent strains resistant to antibiotic treatment has made the picture even more complex. Evidence on preventive treatments to avoid recurrence is unclear. Current guidelines indicate the following antibiotics for the treatment of CDI: metronidazole, vancomycin, and fidaxomycin. This short narrative review provides an overview of CDI, antibiotic resistance, and emerging treatments.

## 1. Introduction

### Clostridioides difficile

**Figure 1:**
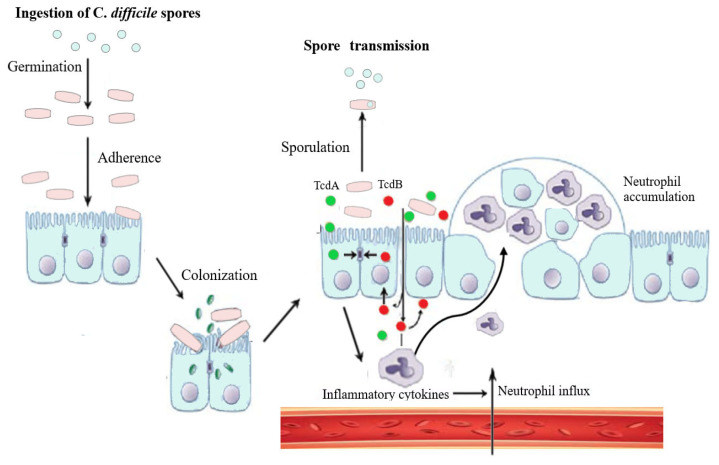
*Clostridioides difficile* life cycle shows dormant spores that germinate into toxin-producing vegetative bacteria. Primary bile acids, synthesised in the liver and secreted into the gastrointestinal (GI) tract, create a favourable environment for germination. Secondary bile acids and metabolic products from commensal bacteria inhibit the growth of vegetative forms. These key metabolites derived from microbes are reduced during antibiotic treatment. The main virulent toxins of the bacterium are toxins A (TcdA) and B (TcdB). TcdA and TcdB cause dysregulation of cellular movement and polarity, microtubule dynamics and vesicle trafficking, and cell cycle progression. Weakening of cell junctions results in neutrophil influx and accumulation.

*Clostridioides difficile* (*C. difficile*) is a Gram-positive bacterium able to produce toxins and spores. In recent years, the incidence of *C. difficile* infection (CDI) has increased both in the context of healthcare-associated infections (HAIs) and community-acquired infections [1]. To date, *C. difficile* is the primary cause of nosocomial infective diarrhoea, and epidemiological data indicate approximately 120,000 cases per year in Europe [2]. A study by Balsells et al. [3] estimated the incidence of CDI at 2.2 per 1000 hospitalisations per year at the global level. Mostly, CDI is associated with prolonged use of clindamycin, third- and fourth-generation cephalosporins, fluoroquinolones, or proton-pump inhibitor (IPP) drugs. Patient risk factors associated with higher incidence of CDI include older age, inflammatory bowel disease, and prolonged or multiple hospitalisations. The clinical symptomatology of CDI includes a broad spectrum of diseases, such as severe diarrhoea, pseudomembranous colitis, fulminant colitis, and toxic megacolon. In addition, a high number of recurrences are often observed [4]. Regarding CDI, antibiotic treatment can be both curative and, paradoxically, a risk factor. A healthy human microbiome and an efficient host immune system are the main defence mechanisms against CDI, and, in most cases, are sufficient to avoid complications. However, an intestinal dysmicrobiome following prolonged antibiotic therapy may represent a breeding ground for bacterial colonisation and clinical symptoms associated with severe CDI [5]. Especially, treatment with broad-spectrum antibiotic causes gut dysbiosis and survival of resistant *C. difficile* bacteria. The fundamental role of the human microbiota is to hinder CDI through nutritional competition and the production of antimicrobial peptides and metabolites [6]. Variations in the diversity of human microbiota have been associated with susceptibility to and recurrence of CDI [7]. The toxins produced by *C. difficile* cause the destruction of the cell cytoskeleton, epithelial cell death, and weakening of cell junction proteins, resulting in pseudomembranous colitis and diarrhoea [8]. Its main virulent toxins are toxins A (TcdA) and B (TcdB). Both TcdA and TcdB target the G-protein family of signalling proteins called Rho GTPases (e.g., RhoA, Rac1, Ras, and Cdc42), which regulate cellular movement and polarity, microtubule dynamics and vesicle trafficking, and cell cycle progression [1] (Figure 1).

TcdB is thought to be more involved in the pathogenesis and symptomatology of CDI than TcdA. However, both toxins can cause damage to the intestinal mucosa [8]. The relationship between antibiotic treatment and CDI is complex. On the one hand, the prolonged and inappropriate use of antibiotics is a major risk factor for CDI; on the other hand, the use of antibiotics, particularly broad-spectrum ones such as cephalosporins or clindamycin, alters the endogenous intestinal microbiota, facilitating the colonisation of the GI tract by *C. difficile* [9]. To date, antibiotics represent the main therapeutic option available for CDI. Treatment of non-severe CDI involves discontinuing antibiotic therapy, if possible, and administering fidaxomicin 200 mg twice daily [10]. 

When fidaxomicin is not administrable, oral vancomycin 125 mg four times per day for 10 days is recommended [10]. In case of an episode of CDI with increased risk of recurrence, bezlotoxumab can be added to the oral standard of care treatment [11].

For the treatment of severely complicated CDI, the standard of care includes vancomycin 125 mg four times per day for 10 days or fidaxomycin 200 mg twice a day for 10 days [12]. When a patient deteriorates or progresses to severe CDI while on antibiotic therapy, the addition of intravenous tigecycline 50 mg twice daily (100 mg loading dose) may be considered on a case-by-case basis [13].

The objectives of this research study are to highlight the emerging public health problem of resistant and increasingly relapsing forms of CDI [13] and to describe the latest evidence on pharmacological treatments. In addition, we discuss the factors contributing to the difficulty of clearly determining the true burden of antimicrobial resistance in *C. difficile* and how this affects the treatment of CDI.

## 2. Antimicrobial Resistance and CDI

Antimicrobial resistance (AMR) affects CDI in several ways. Multiresistant bacterial infections may require antibiotic treatment for long periods, increasing the risk of developing CDI. In addition, the continued use of antibiotics is probably the cause of the emergence of increasingly virulent and persistent bacterial forms of *C. difficile*, such as ribotype 027, which is known as a significant predictor of severe CDI and mortality [14]. Antibiotic resistance in CDI is constantly evolving as new resistance-determining mechanisms emerge. The prevalence of diverse CDI strains shows distinct geographical patterns in the world. Understanding the epidemiology of antibiotic resistance is important for effective AMR surveillance and for reducing the spread of resistance determinants between different strains of CDI and also between diverse microbial species. An antibiotic stewardship intervention that restricted the use of fluoroquinolones, clindamycin, amoxicillin/clavulanate, and cephalosporins was effective in reducing the epidemic ribotypes, e.g., 001 and 027 [9]. As the number of antibiotics available for the treatment [15] of CDI is limited, information on the resistance of circulating *C. difficile* strains is crucial. A recent meta-analysis that considered 111 studies showed that metronidazole, vancomycin, fidaxomicin, meropenem, and piperacillin/tazobactam rarely reported resistance to *C. difficile*, and that fluoroquinolones and clindamycin were the antimicrobials at high risk for promoting the development of CDI and resistance [16]. Teng and colleagues [17] showed that all the antibiotic classes considered were at risk for CDI, and lincosamides (e.g., clindamycin), monobactams, penicillin combinations, and carbapenems were the antibiotic classes with the highest association with CDI. *C. difficile* resistance to metronidazole and vancomycin was low compared to other drugs used to treat CDI [18]. Another recent study collected 75 *C. difficile* isolates (time interval 1980–1986) in the United Kingdom (UK) and tested their susceptibility to a panel of 16 antimicrobials to be compared to 416 *C. difficile* isolates detected in the last decade (time interval 2012–2016). The study concluded that *C. difficile* resistance had increased for all classes of antibiotics in the UK since 1980 [19]. In addition, another study collected 593 *C. difficile* isolates between 2012 and 2017, showing that elevated minimum inhibitory concentrations (MICs) of antibiotics used for the treatment of CDI were rare, with no increase in MICs during this reference period [20]. An epidemiological survey involving 22 European countries [21] considered 953 isolated *C. difficile* ribotypes and their susceptibility to antibiotic treatment. The most frequently isolated ribotypes were 027, 014, 001/072, and 078, as in previous European studies, with evidence neither of resistance to fidaxomicin nor of reduced susceptibility to metronidazole and vancomycin. On the contrary, multiple ribotypes [16] showed resistance to rifampin, moxifloxacin, and clindamycin (13%, 40%, and 50% of total isolates, respectively). Table 1 summarized the main studies on resistant CDI published in PubMed from 2011 to 2023.

## 3. New Treatments for CDI

AMR also has a high impact on CDI. In recent years, increasingly virulent strains that are resistant to common antibiotic treatments have emerged. Therefore, it is imperative that new antibiotics directed against *C. difficile* are available, and alternative therapeutic strategies must be implemented. In particular, new therapeutics may be useful in avoiding relapses.

Phage therapy appears to be an important weapon in the treatment of CDI. The antibacterial method using bacteriophages involves viruses that infect bacteria with bactericidal effects as an alternative treatment to antibiotics. To date, many phages specific to *C. difficile* have been identified. A great deal of evidence generated over the past ten years has shown that the application of phages as monotherapy [22,23] targeted to *C. difficile* should be considered as a viable therapeutic alternative. However, all these studies have not provided an effective treatment for CDI. Another line of research involves endolysin [24], a bacteriophage enzyme, to hydrolyse the bacterial cell wall. Recently, next-generation phage therapy based on metagenomic information [25] has emerged as an effective method to obtain genomic information on bacterial–host phage associations and to identify new endolysin sequences. Therefore, phage therapy against CDI appears promising, even if phages with *C. difficile* as the host are not identified easily.

The use of monoclonal antibodies can be an alternative strategy to antibiotics for CDI treatment. Bezlotoxumab is a monoclonal antibody already used in clinical practice, indicated for the prevention of recurrent CDI (rCDI) as it provides passive immunity against the toxin produced by the development of persistent or newly acquired *C. difficile* spores. It binds with high affinity to *C. difficile* toxin B, neutralising its activity. Bezlotoxumab was tested in adults affected by primary or rCDI in two global phase 3 studies (MODIFY I and MODIFY II), resulting in a significant reduction in the rate of rCDI compared to placebo (17% vs. 28% in MODIFY I and 16% vs. 26% in MODIFY II; *p* < 0.001) [26]. To date, the use of bezlotoxumab to reduce CDI recurrence is included in major international guidelines [27,28].

Faecal microbiota transplantation (FMT) consists of the infusion of faeces from a healthy donor to the GI tract of a recipient patient in order to treat a specific disease associated with an alteration of the gut microbiota, e.g., ulcerative colitis (UC) and metabolic syndrome (MS) [29]. Numerous pieces of evidence have clearly demonstrated that FMT is a highly effective treatment against rCDI [30]. Based on these data, both the European Society of Microbiology and Infectiology and the American College of Gastroenterology recommend FMT as a treatment for rCDI [31,32]. FMT reduces the risk of rCDI through the restoration of the microbiome. However, to date, little clinical evidence generated from well-structured studies has been reported, limiting the current knowledge on the full efficacy and safety of FMT [33]. Prolonged treatment with antibiotics targeting CDI may still aggravate the human microbiome by representing one of the main factors in changing the composition and function of the microbiome. Restoration of the homeostasis of the microbiome is essential for lasting clinical resolution and avoidance of recurrence. From this perspective, treatment with FMT may have potential benefits. Case series reports have supported efficacy estimates up to 93% [34]. A systematic review and meta-analysis of 45 studies showed a significant overall clinical effect at week 8 after repeated (89–94% in 24 studies including 1855 patients) or single FMT (80–88% in 43 studies including 2937 patients) [2]. The best method of administration was lower GI endoscopy compared to the other methods considered. Ultimately, FMT is effective for rCDI depending on the method and number of administrations [2,26]. An extensive Cochrane research evaluated the benefits and disadvantages of FMT for the treatment of rCDI in immunocompetent individuals [35]. The study showed that in immunocompetent adults with rCDI, FMT probably leads to a marked increase in the resolution of rCDI compared to alternative treatments such as antibiotics. However, the study did not demonstrate an effect of FMT in immunocompromised individuals [35]. FMT or bezlotoxumab in addition to standard antibiotics are preferred for the treatment of second or further rCDI in the main guidelines [27].

Due to the increasing incidence and difficulty of treating rCDI, it is becoming a major clinical and health economics issue. The identification of risk factors and the best therapeutic treatments to avoid recurrences are crucial targets. Advanced age, use of antibiotics, gastric acid suppression with proton-pump inhibitors, and infection with hypervirulent and resistant strains are currently considered the main risk factors for rCDI. The use of monoclonal antibodies or FMTs is of enormous importance in reducing the risk of rCDI. The human gut microbiota consists of a huge and complex community of microorganisms. Continuous treatment with antimicrobial chemotherapeutics can reduce the diversity of the gut microbiota; indeed, it has been shown that the faecal microbiota of patients with rCDI is variable in bacterial composition and characterised by a marked decrease in species. FMT can restore the variability of the gut’s bacterial composition and improve rCDI symptoms.

The evidence on the most suitable treatments for the prevention of primary *C difficile* infections is currently unclear. Among the various classes of antibiotics, intravenously administered β-lactams are considered to be at high risk for favouring CDI as they are excreted in the GI tract with consequent alteration of the gut microbiome. The degradation of antibiotics in the upper GI tract by oral administration of β-lactamases together with β-lactam antibiotics may be effective in reducing the risk of CDI. A randomised, double-blind, phase 2b study showed that oral administration of β-lactamase together with intravenous ceftriaxone for the treatment of pulmonary infections reduced the risk of CDI compared to placebo [36].

Considering that *C. difficile* uses faecal–oral transmission as its main route, a mucosal vaccine generating IgA and IgG responses could prevent colonisation and disease. Vaccines against TcdA and TcdB, administered parenterally, are in clinical trials [37,38]. Since the onset of CDI involves the mucosa colonization to cause disease, an oral mucosa vaccine represents a potential preventive strategy [39,40,41]. In the lamina propria of the intestinal mucosa, in addition to connective tissue, there are cells of the immune system such as lymphocytes, granulocytes, and lymphoid tissue (sometimes grouped into nodules called Peyer plaques) that defend the organism from foreign agents. A number of T cells in the lamina can be stimulated by resident macrophages and dendritic cells (DCs). In addition, gut-associated lymphoid tissue (GALT), via microfold (M) cells, processes natural or vaccine antigens and induces humoral immune responses [42,43]. The main disadvantage of passive vaccination of the oral mucosa is the strong dependence on the pharmaceutical technology used, i.e., orally administered antibodies can be degraded in the GI tract before reaching the target site with a possible reduction in the effectiveness of vaccination [44,45]. Some clinical evidence has reported that passive oral administration of anti-TcdA and anti-TcdB may prevent rCDI. One study showed complete protection against CDI relapses in 16 patients over a period of 333 days without reporting any serious adverse events [46].

An oral microbiome therapy indicated to avoiding rCDI showed efficacy and safety. The microbiome therapeutic is a mixture of purified Firmicutes spores. A phase 3 trial with 182 participants showed that in patients with symptom resolution from CDI after treatment with standard antibiotics, oral administration of SER-109 reduced the risk of recurrent infections better than placebo, with an excellent safety profile [47,48]. The US Food and Drug Administration (FDA) has authorised microbiota-based therapy to prevent relapses in adults after antibacterial treatment for rCDI. The clinical evidence leading to authorisation was generated in the ECOSPOR III and ECOSPOR IV studies. ECOSPOR III showed that oral microbiota therapy reduced CDI relapses at 8 weeks after treatment, with around 88% of subjects free of relapse versus 60% of participants who received the placebo. The ECOSPOR IV pre-registration study generated important evidence of efficacy. ECOSPOR IV was an open-label study that evaluated 263 adults with rCDI [40]. The results showed that 91% of the subjects were relapse-free 8 weeks after treatment, while 86% were relapse-free 24 weeks after treatment [40]. In these studies, oral microbiota therapy was well tolerated even in individuals with comorbidities. Most adverse events affected the GI tract with mild to moderate effects. These results support an important role of SER-109 in the clinical management of rCDI. RBX2660 is a commercially available microbiota-based live biotherapeutic for rCDI treatment. RBX2660 consists of a single 150 mL pre-packaged dose comprising a ready-to-use microbiota suspension containing approximately 107 different live organisms/mL, including Bacteroidetes and Firmicutes. RBX2660 acts by repopulating and restoring the gut microbiome 7 days after treatment and up to 24 months [49,50,51]. The safety and efficacy of RBX2660 were demonstrated in two multicentre and randomised studies: the pivotal PUNCH CD3 clinical trial and the phase 2b PUNCH CD2 study. In the latter, results demonstrated a treatment success rate of 78.9%. Among patients who responded to treatment with RBX2660, 97% remained rCDI-free at 6 months, while 95% and 91% remained event-free at 12 and 24 months, respectively. In the PUNCH CD2 study, treatment efficacy was evaluated after two doses of RBX2660 compared to two doses of placebo; when evaluating relapse prevention (no diarrhoea for 8 weeks), a significant improvement in efficacy was found for one dose of RBX2660 compared to placebo, with treatment success in 87.5% of the group treated with a single dose of RBX2660 compared to 58.1% of the placebo group [49,50,51]. Table 2 summarized the main evidence the on innovative treatments against CDI published in PubMed from 2011 to 2023.

## 4. Discussion

In the past decade, CDI has become one of the most detrimental nosocomial infections. Cases of CDI have increased in number and severity, mainly due to the emergence of hypervirulent strains resistant to available antimicrobials. Certainly, one of the most important actions to be implemented is prevention, with healthcare workers being educated on preventive measures such as hand washing and proper decontamination of medical devices and the patient’s environment. The excessive and inappropriate use of antimicrobial chemotherapeutics, coupled with sub-optimal health surveillance of cases of infection, have probably fuelled the development of this urgent health problem. Acquired antimicrobial resistance by *C. difficile* is multifactorial and caused by resistance genes leading to alterations in the antimicrobial target and biofilm formation [47]. In combination with all preventive measures, an important action is the optimal management of antibiotics. Elderly patients who are hospitalised and treated with antibiotics are at higher risk of rCDI. FMT is a very promising treatment of rCDI. Key actions recommended to counter rCDI include the appropriate use of pharmacological agents according to the right dose and timing indicated, especially in case of broad-spectrum antibiotics, and strict implementation of infection prevention and control (IPC) measures. To date, the main international guidelines suggest the use of fidaxomycin and vancomycin for the treatment of CDI, as they are still effective. New therapeutic options are needed to counter the increasing incidence and severity of *C. difficile* cases. Promising new therapeutic alternatives to the use of antimicrobial chemotherapeutics are either in trials or in the early stages of commercialisation; among them, therapeutics for the restoration of human gut microbiota [47,48,49,50,51].

### Future Directions

Several research avenues can be pursued to combat CDI and *C. difficile* resistant strains [52]. For example, the *C. difficile* sporulation pathway can be a possible therapeutic molecular target, as it is necessary for the transmission and persistence of the disease [49]. Another therapeutic target is the spore germination pathway. Recent evidence has shown new antimicrobial compounds with bactericidal activity against vegetative cells of *C. difficile* and inhibition of spore germination [53]. Moreover, direct competition with a non-toxic *C. difficile* strain (NTCD) can be used as preventive strategy of CDI. In vitro models of the human gut have shown that inoculation with an NTCD successfully prevents the development of CDI with the hypervirulent strain RT027 after the administration of a number of different antibiotics [54].

## 5. Conclusions

In recent years, infections caused by *C. difficile* have increased in terms of the incidence and severity of cases, both in the healthcare setting and in the community. Because of this growing threat, new innovative therapeutic strategies are needed to prevent epidemics. Current therapies for rCDI do not address the problem of intestinal dysmicrobism, which promotes the germination of *C. difficile* spores in toxin-producing bacteria. In this direction, evidence to date indicates that treatment with FMT is helpful in restoring the human microbiome and reducing rCDI with excellent efficacy and safety. Innovative therapeutic strategies are urgently needed to protect the human microbiome while reducing the risk of CDI relapses. Several new therapeutic strategies are currently being tested. While evidence demonstrates the strengths and weaknesses of these new therapies, further well-structured research will allow us to fully define the efficacy and safety profile of each innovative care modality.

## Figures and Tables

**Table 1:** Main studies showing antimicrobial resistance of circulating strains of *Clostridioides difficile* to antibiotic treatment, 2011–2023.

Evidence	Reference
Fluoroquinolones and clindamycin developed a high level of resistance.	Sholeh M, 2020 [16]
Lincosamides, monobactams, penicillin combinations, and carbapenems were the antibiotic classes with the highest association with CDI.	Teng C, 2019 [17]
Resistance of *C. difficile* to metronidazole and vancomycin was low.	Dilnessa T, 2022 [18]
*C. difficile* resistance increased for all classes of antibiotics in the UK since 1980.	Jon JV, 2021 [19]
Elevated MICs of antibiotics used for the treatment of CDI were rare, with no increase in MICs over time.	Gargis AS, 2017 [20]
There was no evidence of resistance to fidaxomycin, metronidazole, and vancomycin. Resistance to rifampin, moxifloxacin, and clindamycin was evident in multiple ribotypes.	Freeman J, 2015 [21]

CDI (*Clostridioides difficile* infection); MIC (minimal inhibiting concentration).

**Table 2:** Main evidence on the management of new treatments against *Clostridioides difficile* infection, 2011–2023.

Innovative Treatment	Evidence	Reference
Phage therapy	Phage φCD38-2 is able to infect several isolates of the hypervirulent epidemic strain NAP1/027, which caused severe outbreaks in North America and Europe.	Sekulovic O, 2011 [23]
	The oral delivery of optimised phage combinations resulted in reduced *C. difficile* colonisation at 36 h post infection.	Nale JY, 2016 [22]
Bezlotoxumab	The addition of bezlotoxumab to antibiotic treatment resulted in significant reductions in the rate of rCDI compared to placebo (17% vs. 28% in MODIFY I and 16% vs. 26% in MODIFY II; *p* < 0.001).	Wilcox MH, 2017 [26]
Faecal microbiota transplantation (FMT)	Among 45 studies considered, the overall clinical effect at week 8 was 91% (95% CI: 89–94%, I^2^ = 53%) after repeated FMT (24 studies, 1855 patients) and 84% (80–88%, I^2^ = 86%) after single FMT (43 studies, 2937 patients).	Baunwall SMD, 2020 [34]
	As of 31 March 2022, data from randomised controlled trials showed that FMT resulted in a large increase in the resolution of rCDI in immunocompetent adults compared to alternative treatments, including antibiotics. However, short- and long-term safety need further assessment.	Minkoff NZ, 2023 [35]
Vaccination	Choice of passive immunotherapies or active vaccination modules the efficacy of CDI prevention.	Campidelli C, 2024 [41]
Oral microbiome	In patients with CDI symptom resolution after treatment with standard antibiotics, oral administration of SER-109 was superior to placebo in reducing the risk of recurrent infections, with an excellent safety profile.	Feuerstadt P, 2022 [47]
	RBX2660 restores the gut microbiome 7 days after treatment and up to 24 months.	Chopra T, 2023 [49].

FMT (faecal microbiota transplantation); CDI (*Clostridioides difficile* infection); rCDI (recurrent CDI).

## Data Availability

Not applicable.
